# 6-Iodo-2-methyl-1,3-benzothia­zole

**DOI:** 10.1107/S1600536811004570

**Published:** 2011-02-12

**Authors:** Marijana Đaković, Helena Čičak

**Affiliations:** a Department of Chemistry, Faculty of Science, University of Zagreb, Horvatovac 102a, HR-10000 Zagreb, Croatia

## Abstract

The title compound, C_8_H_6_INS, is essentially planar, the largest deviation from the mean plane being for the I atom [0.075 (3) Å]. The crystal structure is mainly stabilized by inter­molecular C—I⋯N halogen bonds, forming zigzag supra­molecular chains in [10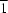]. Relatively short off-set π–π contacts [centroid–centroid distance = 3.758 (2) Å] between the thia­zole rings of inversion-related mol­ecules link neighbouring chains and provide the secondary inter­actions for building the crystal structure.

## Related literature

For the application of benzothia­zoles as biologically active compounds, see: Leong *et al.* (2004 ▶); Yildiz-Oren *et al.* (2004 ▶); Lockhart *et al.* (2005 ▶); Sheng *et al.* (2007 ▶). For the synthesis of the title compound, see: Racané *et al.* (2006 ▶, 2011 ▶). For related 1,3-benzothia­zole structures, see: Matković-Čalogović *et al.* (2003 ▶); Pavlović *et al.* (2009 ▶); Đaković *et al.* (2009 ▶); Čičak *et al.* (2010 ▶). For graph-set theory, see: Etter (1990 ▶); Bernstein *et al.* (1995 ▶). For a description of the Cambridge Structural Database, see: Allen (2002 ▶).
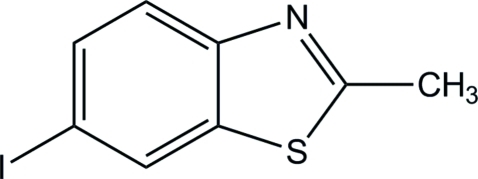


## Experimental

### 

#### Crystal data

C_8_H_6_INS
*M* *_r_* = 275.11
Monoclinic, 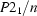

*a* = 8.3255 (3) Å
*b* = 7.6967 (3) Å
*c* = 13.8083 (5) Å
β = 90.686 (4)°
*V* = 884.76 (6) Å^3^
*Z* = 4
Mo *K*α radiation
μ = 3.79 mm^−1^
*T* = 296 K
0.47 × 0.38 × 0.14 mm

#### Data collection

Oxford Diffraction Xcalibur diffractometer with a Saphire-3 CCD detector
Absorption correction: multi-scan (*CrysAlis PRO*; Oxford Diffraction, 2009 ▶) *T*_min_ = 0.253, *T*_max_ = 0.658
13190 measured reflections
1928 independent reflections
1729 reflections with *I* > 2σ(*I*)
*R*_int_ = 0.027

#### Refinement

*R*[*F* ^2^ > 2σ(*F* ^2^)] = 0.024
*wR*(*F* ^2^) = 0.064
*S* = 1.06
1928 reflections
101 parameters
H-atom parameters constrained
Δρ_max_ = 0.84 e Å^−3^
Δρ_min_ = −0.72 e Å^−3^

### 

Data collection: *CrysAlis PRO* (Oxford Diffraction, 2009 ▶); cell refinement: *CrysAlis PRO*; data reduction: *CrysAlis PRO*; program(s) used to solve structure: *SHELXS97* (Sheldrick, 2008 ▶); program(s) used to refine structure: *SHELXL97* (Sheldrick, 2008 ▶); molecular graphics: *ORTEP-3* (Farrugia, 1997 ▶) and *Mercury* (Macrae *et al.*, 2006 ▶); software used to prepare material for publication: *SHELXL97* and *PLATON* (Spek, 2009 ▶).

## Supplementary Material

Crystal structure: contains datablocks global, I. DOI: 10.1107/S1600536811004570/fj2389sup1.cif

Structure factors: contains datablocks I. DOI: 10.1107/S1600536811004570/fj2389Isup2.hkl

Additional supplementary materials:  crystallographic information; 3D view; checkCIF report

## Figures and Tables

**Table 1:** Halogen-bond geometry (Å, °)

	C4—I1	I1⋯N1^i^	C4⋯N1^*i*^	C4—I1⋯N1^i^
C4—I1⋯N1^*i*^	2.103 (3)	3.158 (2)	5.257 (4)	175.99 (9)

Symmetry code: (i) 
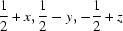
.

**Fig. 1.**
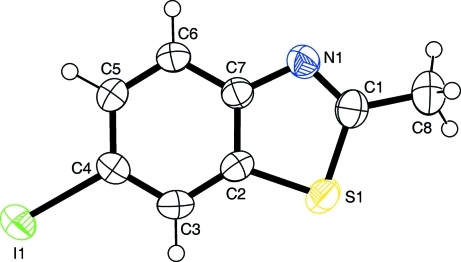
Molecular structure of the title compound with the atom labeling scheme. Displacement ellipsoids for non-H atoms are drawn at the 50% probability level.

**Fig. 2.**
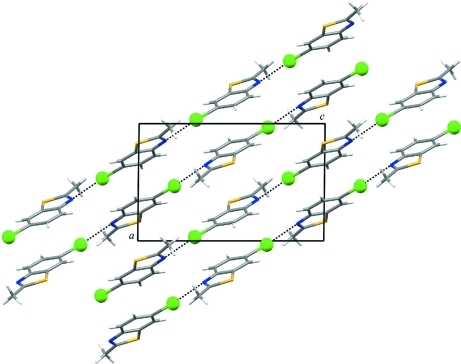
Crystal packing of the title compound viewed down the b axis showing halogen bonds as dashed lines.

**Fig. 3.**
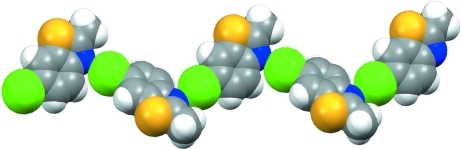
Spacefill representaton of a zigzag halogen bonding chain running in [1 0 - 1].

## supplementary crystallographic information

### Comment

This work was a part of our preparative, structural, mechanistic and computational investigation of a series of substituted benzothiazoles (bta), which attract considerable interest due to their biological activities.

The molecule is almost ideally planar (r.m.s. deviation = 0.009 Å), with the largest deviation from the plane being that of atom I1 [0.075 (3) Å] (Fig.1). The geometry of the benzothiazole rings is consistent with other 1,3-benzothiazoles listed in the CSD base (Allen *et al.*, 2002). The two S—C bonds of the thiazole ring [S1—C1 and S1—C2] differ with respect to each other, but both are within two bortherline cases, single S—C [1.82 Å] and double S=C [1.56 Å], while the endocyclic C—N bond is dominantly double in character. The differences in C—C bonds within benzene ring are common for such fused rings.

In the crystal structure halogen bonds are the principal specific interactions responsible for the crystal packing. There is only one short and directional C—I···N contact [C—I = 2.103 (3) Å] (see Table 1) that link the molecules into antiparallel zigzag C(7) chains (Etter, 1990; Bernstein *et al.*, 1995) in [1 0 - 1] direction (Figs. 2 and 3).

Relatively short off-set π–π contacts [*C*g···*C*g = 3.758 (2) Å] between the thiazole rings, belonging to the molecules that are related by an inversion centre, link the neighboring supramolecular chains and provide the secondary interactions for building the crystal structure.

The structure of the title compound is one more example showing that halogen bonding is also as effective and reliable tool for assembling molecules into supramolecular architectures.

### Experimental

Colourless single crystals of the title compound were obtained by slow evaporation of a dichloromethane solution.

### Refinement

All H atoms were placed in geometrically idealized positions and constrained to ride on their parent C atom at distances of 0.93 or 0.96 Å for aromatic or methyl H atoms, respectively, and with *U*_iso_(H) = 1.2*U*_eq_(C) (for aromatic H) or *U*_iso_(H) = 1.5*U*_eq_(C) (for methyl group).

### Crystal data

**Table:**

C_8_H_6_INS	*F*(000) = 520
*M**_r_* = 275.11	*D*_x_ = 2.065 Mg m^−^^3^
Monoclinic, *P*2_1_/*n*	Mo *K*α radiation, λ = 0.71073 Å
Hall symbol: -P 2yn	Cell parameters from 10010 reflections
*a* = 8.3255 (3) Å	θ = 4.4–32.6°
*b* = 7.6967 (3) Å	µ = 3.79 mm^−^^1^
*c* = 13.8083 (5) Å	*T* = 296 K
β = 90.686 (4)°	Plate, colourless
*V* = 884.76 (6) Å^3^	0.47 × 0.38 × 0.14 mm
*Z* = 4	

### Data collection

**Table:**

Oxford Diffraction Xcalibur diffractometer with a Saphire-3 CCD detector	1928 independent reflections
Radiation source: Enhance (Mo) X-ray Source	1729 reflections with *I* > 2σ(*I*)
graphite	*R*_int_ = 0.027
Detector resolution: 16.3426 pixels mm^-1^	θ_max_ = 27.0°, θ_min_ = 4.6°
CCD scans	*h* = −10→10
Absorption correction: multi-scan (*CrysAlis PRO*; Oxford Diffraction, 2009)	*k* = −9→9
*T*_min_ = 0.253, *T*_max_ = 0.658	*l* = −17→17
13190 measured reflections	

### Refinement

**Table:**

Refinement on *F*^2^	Primary atom site location: structure-invariant direct methods
Least-squares matrix: full	Secondary atom site location: difference Fourier map
*R*[*F*^2^ > 2σ(*F*^2^)] = 0.024	Hydrogen site location: inferred from neighbouring sites
*wR*(*F*^2^) = 0.064	H-atom parameters constrained
*S* = 1.06	*w* = 1/[σ^2^(*F*_o_^2^) + (0.0397*P*)^2^ + 0.4162*P*] where *P* = (*F*_o_^2^ + 2*F*_c_^2^)/3
1928 reflections	(Δ/σ)_max_ = 0.001
101 parameters	Δρ_max_ = 0.84 e Å^−^^3^
0 restraints	Δρ_min_ = −0.72 e Å^−^^3^

### Special details

**Table:**

Experimental. Solvent used: CH_2_Cl_2_ Crystal mount: glued on a glass fibre Mosaicity (°): 1.1 (1) Frames collected: 892 Seconds exposure per frame: 5 Degree rotation per frame: 1.0 Crystal-Detector distance (mm): 50.0.
Geometry. Bond distances, angles *etc*. have been calculated using the rounded fractional coordinates. All su's are estimated from the variances of the (full) variance-covariance matrix. The cell e.s.d.'s are taken into account in the estimation of distances, angles and torsion angles
Refinement. Refinement on *F*^2^ for ALL reflections except those flagged by the user for potential systematic errors. Weighted *R*-factors *wR* and all goodnesses of fit *S* are based on *F*^2^, conventional *R*-factors *R* are based on *F*, with *F* set to zero for negative *F*^2^. The observed criterion of *F*^2^ > σ(*F*^2^) is used only for calculating -*R*-factor-obs *etc*. and is not relevant to the choice of reflections for refinement. *R*-factors based on *F*^2^ are statistically about twice as large as those based on *F*, and *R*-factors based on ALL data will be even larger.

### Fractional atomic coordinates and isotropic or equivalent isotropic displacement parameters (Å^2^)

**Table:**

	*x*	*y*	*z*	*U*_iso_*/*U*_eq_	
I1	0.96733 (2)	0.14994 (3)	0.30624 (1)	0.0485 (1)	
S1	0.66879 (11)	0.75187 (9)	0.49021 (6)	0.0518 (3)	
N1	0.6453 (3)	0.5615 (3)	0.64355 (18)	0.0447 (8)	
C1	0.6117 (4)	0.7135 (4)	0.6097 (2)	0.0457 (9)	
C2	0.7440 (3)	0.5425 (3)	0.4842 (2)	0.0392 (8)	
C3	0.8155 (3)	0.4585 (4)	0.40718 (19)	0.0430 (8)	
C4	0.8633 (3)	0.2887 (4)	0.42068 (19)	0.0399 (8)	
C5	0.8427 (4)	0.2058 (4)	0.5097 (2)	0.0452 (9)	
C6	0.7728 (4)	0.2900 (4)	0.5857 (2)	0.0467 (9)	
C7	0.7211 (3)	0.4614 (3)	0.5734 (2)	0.0386 (7)	
C8	0.5343 (5)	0.8546 (4)	0.6669 (3)	0.0600 (11)	
H3	0.83070	0.51470	0.34840	0.0520*	
H5	0.87710	0.09160	0.51740	0.0540*	
H6	0.75980	0.23380	0.64480	0.0560*	
H8A	0.46780	0.80420	0.71580	0.0900*	
H8B	0.46950	0.92540	0.62460	0.0900*	
H8C	0.61580	0.92510	0.69710	0.0900*	

### Atomic displacement parameters (Å^2^)

**Table:**

	*U*^11^	*U*^22^	*U*^33^	*U*^12^	*U*^13^	*U*^23^
I1	0.0488 (1)	0.0567 (2)	0.0402 (1)	0.0012 (1)	0.0048 (1)	−0.0055 (1)
S1	0.0658 (5)	0.0376 (4)	0.0518 (4)	0.0063 (3)	−0.0035 (3)	0.0058 (3)
N1	0.0487 (13)	0.0419 (13)	0.0437 (13)	−0.0022 (10)	0.0082 (10)	−0.0014 (10)
C1	0.0416 (14)	0.0411 (14)	0.0543 (17)	−0.0019 (12)	−0.0027 (12)	−0.0054 (12)
C2	0.0424 (14)	0.0345 (12)	0.0407 (13)	−0.0029 (11)	−0.0035 (11)	0.0049 (11)
C3	0.0482 (15)	0.0457 (14)	0.0351 (13)	−0.0040 (12)	−0.0001 (11)	0.0075 (12)
C4	0.0393 (14)	0.0457 (14)	0.0347 (13)	−0.0025 (11)	0.0025 (11)	−0.0027 (11)
C5	0.0536 (16)	0.0347 (13)	0.0474 (16)	0.0044 (12)	0.0043 (13)	0.0030 (11)
C6	0.0593 (18)	0.0394 (13)	0.0415 (15)	−0.0005 (13)	0.0114 (13)	0.0073 (12)
C7	0.0396 (13)	0.0364 (12)	0.0399 (13)	−0.0035 (11)	0.0037 (10)	0.0013 (10)
C8	0.062 (2)	0.0511 (19)	0.067 (2)	0.0089 (15)	−0.0004 (17)	−0.0108 (15)

### Geometric parameters (Å, °)

**Table:**

I1—C4	2.103 (3)	C4—C5	1.397 (4)
S1—C1	1.748 (3)	C5—C6	1.369 (4)
S1—C2	1.731 (2)	C6—C7	1.397 (4)
N1—C1	1.289 (4)	C3—H3	0.9300
N1—C7	1.395 (4)	C5—H5	0.9300
C1—C8	1.494 (5)	C6—H6	0.9300
C2—C3	1.385 (4)	C8—H8A	0.9600
C2—C7	1.396 (4)	C8—H8B	0.9600
C3—C4	1.378 (4)	C8—H8C	0.9600
			
I1···C1^i^	3.826 (3)	H3···H8A^vii^	2.5800
I1···N1^ii^	3.158 (2)	H5···S1^viii^	3.1600
I1···H5^iii^	3.3100	H5···I1^iii^	3.3100
S1···H5^iv^	3.1600	H5···H5^iii^	2.5400
S1···H8B^v^	3.1600	H8A···H3^ix^	2.5800
N1···I1^vi^	3.158 (2)	H8B···S1^v^	3.1600
C1···I1^i^	3.826 (3)		
			
C1—S1—C2	89.46 (14)	N1—C7—C6	125.3 (2)
C1—N1—C7	110.3 (2)	C2—C7—C6	119.0 (2)
S1—C1—N1	115.8 (2)	C2—C3—H3	121.00
S1—C1—C8	120.0 (2)	C4—C3—H3	121.00
N1—C1—C8	124.1 (3)	C4—C5—H5	119.00
S1—C2—C3	129.1 (2)	C6—C5—H5	120.00
S1—C2—C7	108.70 (19)	C5—C6—H6	120.00
C3—C2—C7	122.2 (2)	C7—C6—H6	120.00
C2—C3—C4	117.7 (2)	C1—C8—H8A	110.00
I1—C4—C3	120.06 (19)	C1—C8—H8B	110.00
I1—C4—C5	119.0 (2)	C1—C8—H8C	109.00
C3—C4—C5	120.9 (3)	H8A—C8—H8B	109.00
C4—C5—C6	121.1 (3)	H8A—C8—H8C	109.00
C5—C6—C7	119.1 (3)	H8B—C8—H8C	109.00
N1—C7—C2	115.7 (2)		
			
C2—S1—C1—N1	0.5 (3)	S1—C2—C7—C6	180.0 (2)
C2—S1—C1—C8	178.5 (3)	C3—C2—C7—N1	−179.1 (2)
C1—S1—C2—C3	179.0 (3)	C3—C2—C7—C6	0.4 (4)
C1—S1—C2—C7	−0.6 (2)	C2—C3—C4—I1	178.41 (19)
C7—N1—C1—S1	−0.3 (3)	C2—C3—C4—C5	−1.1 (4)
C7—N1—C1—C8	−178.2 (3)	I1—C4—C5—C6	−178.8 (2)
C1—N1—C7—C2	−0.2 (3)	C3—C4—C5—C6	0.8 (5)
C1—N1—C7—C6	−179.6 (3)	C4—C5—C6—C7	0.2 (5)
S1—C2—C3—C4	−179.0 (2)	C5—C6—C7—N1	178.6 (3)
C7—C2—C3—C4	0.6 (4)	C5—C6—C7—C2	−0.8 (4)
S1—C2—C7—N1	0.5 (3)		

Symmetry codes: (i) −*x*+2, −*y*+1, −*z*+1; (ii) *x*+1/2, −*y*+1/2, *z*−1/2; (iii) −*x*+2, −*y*, −*z*+1; (iv) *x*, *y*+1, *z*; (v) −*x*+1, −*y*+2, −*z*+1; (vi) *x*−1/2, −*y*+1/2, *z*+1/2; (vii) *x*+1/2, −*y*+3/2, *z*−1/2; (viii) *x*, *y*−1, *z*; (ix) *x*−1/2, −*y*+3/2, *z*+1/2.

### Table 1 Halogen-bond geometry (Å, °)

**Table:**

	C4—I1	I1···N1^i^	C4···N1*^i^*	C4—I1···N1^i^
C4—I1···N1*^i^*	2.103 (3)	3.158 (2)	5.257 (4)	175.99 (9)

Symmetry code: (i) 1/2 + *x*, 1/2–*y*, –1/2 + *z*.
